# Prevalence and risk factors for chronic obstructive lung disease in HIV-infected patients in the HAART era

**DOI:** 10.1186/1758-2652-13-S4-P232

**Published:** 2010-11-08

**Authors:** G Madeddu, AG Fois, GM Calia, F Becciu, B Piras, ML Fiori, V Spada, C Lovigu, M Mannazzu, P Pirina, MS Mura

**Affiliations:** 1 University of Sassari, Department of Infectious Diseases, Sassari, Italy; 2 University of Sassari, Department of Respiratory Diseases, Sassari, Italy

## Purpose of the study

To evaluate the prevalence of respiratory symptoms and COPD in a stable HIV-infected outpatient population and to further investigate the role of HAART and other possibly associated risk factors.

## Methods

All participants completed a questionnaire for pulmonary symptoms and the MRC dyspnoea scale. A complete spirometry with evaluation of the residual volume was performed using the bodyplethismographic method. We considered the Total Lung Capacity (TLC), Forced Expiratory Volume (FEV1) at 1st second and the FEV1/FVC ratio (Tiffenau Index) for each patient.

## Summary of results

We enrolled 111 HIV-infected patients with a mean age of 42.3 ± 8.1 years and 65 HIV-negative age and sex-matched controls. Seventy-seven (69.4%) HIV patients were male and 39 (35.1%) were in CDC stage C. Eighty-seven (78.4%) were receiving HAART. Mean CD4 cell count was 541 ± 243 cells/mm^3^ and 79 (71.2%) had an undetectable HIV-RNA. Sixty-three (56.8%) patients were active smokers whereas 48 (43.2%) were non-smokers. No significant difference in age, sex, proportion of smokers and pack-year history of smoking was evidenced between HIV positive patients and controls. However, HIV-infected individuals had significantly lower FEV1 (p=0.002) and FEV1/FVC (p=0.028), whereas TLC was significantly higher (p=0.018). Furthermore, HIV-infected patients had a significantly higher proportion of any respiratory symptom (p=0.002), cough (p=0.006) and dyspnoea (p=0.020). HIV-infected patients had also a significantly (p=0.008) higher proportion (23.4%) of COPD in respect of HIV-negative controls (7.7%). In a multivariate regression analysis significant predictors of respiratory symptoms were current smoking (AOR 11.18; 95% C.I 3.89-32.12) and previous bacterial pneumonia (AOR 4.41; 95% C.I. 1.13-17.13), whereas the only statistically significant predictor of COPD was current cigarette smoking (AOR 5.94; 95% C.I. 1.77-19.96). HAART receipt was not significantly associated with respiratory symptoms nor with COPD.

## Conclusions

our results suggest a role for HIV infection itself and for current cigarette smoking in the development of respiratory symptoms and COPD in HIV-infected patients. HAART did not seem to reduce the risk of respiratory symptoms and COPD, in our cases. Thus, our results suggest that HIV-infected patients should be screened for chronic respiratory disease in order to early identify those at risk or those who need specific treatment.

